# Supplementary material for: Inhibition of Dermatophyte Fungi by Australian Jarrah Honey
Source: Pathogens. 2021 Feb 11;10(2):194. doi: 10.3390/pathogens10020194 (PMC7918412; doi:10.3390/pathogens10020194)
Supplement: Supplementary file 1 [file pathogens-10-00194-s001.pdf]

Table S1. Estimated concentrations of H_2O_2 in Jarrah (Barnes 10+) honey dilution series based on the HRP assay

Honey concentration [%(w/v)]	H_2O_2 (μM)
25	448
12.5	224
6.25	112
3.13	56
1.56*	28
0.78	14
0.39	7
0.2	3.5
0.1	1.75

*MIC for *T. rubrum*
